# Eco-social policies, capitalism and the horizon of emancipatory politics

**DOI:** 10.1177/02610183241262733

**Published:** 2024-08-01

**Authors:** FRANCESCO LARUFFA

**Affiliations:** 27212 University of Geneva, Switzerland

**Keywords:** capitalism, eco-social policies, social-ecological transformation, socialism, sustainable welfare

## Abstract

Within the space of a few years, eco-social policies evolved from being endorsed by a marginal community of heterodox scholars to being established in mainstream circles. While this is a welcome development, it also raises questions among critical social policy scholars. How should an emancipatory eco-social policy look? Rather than specifying concrete policies, this article contributes to answering this question by discussing the relationship between eco-social policies and capitalism. The discourse of ‘sustainable welfare’ tends to problematise growth rather than capitalism, risking a technocratic co-optation by neoliberal elites. Recent attempts to provide empirically applicable ‘non-normative’ definitions of eco-social policies risk weakening the critical potential of sustainable welfare ideas, de-politicising global capitalism with its inherent inequalities and unsustainability. I argue that critical scholars should embrace a democratic, feminist, and anti-racist/anti-colonial eco-socialism as the normative horizon of eco-social policies; discuss challenges related to the realisation of this ideal; and propose an agenda for critical eco-social policy research.

## Introduction

There is a rapidly growing volume of literature on eco-social policy. This scholarship is based on the recognition that ecological and social issues need to be framed together. One important reason for this is that environmental policies risk hitting most negatively already disadvantaged groups (e.g., Jenks and Obringer, 2020). Scholars have examined eco-welfare regimes (Koch and Fritz, 2014; Zimmermann and Graziano, 2020); investigated the public support for eco-social policies (e.g., Gugushvili and Otto, 2023); and discussed the merits and shortcomings of various eco-social policy proposals, such as universal basic income and universal basic services (Büchs, 2021a; Coote, 2022), participatory income (McGann and Murphy, 2023) and the re-orientation of activation for eco-social purposes (Dukelow, 2022).

Although it is often framed as a newly emerging research field, its origins can be tracked back to the 1990s – and indeed one of the first contributions on this subject was published by Tony Fitzpatrick (1998) in this Journal. However, it is remarkable that, within the space of a few years, the idea of eco-social policies evolved from being endorsed by a marginal community of heterodox scholars to being widely accepted in mainstream social policy circles, e.g., with various dedicated sessions at conferences such as those of the European Network for Social Policy Analysis (ESPAnet). Of course, this is a welcome development: with the deepening of the ecological crisis, it is essential that welfare scholars study the relationship between the promotion of social and ecological goals. However, the recent progress also raises questions among critical social policy scholars, as the mainstreaming of eco-social policy ideas runs the risk that the approaches that come to dominate this growing field are those that have the least emancipatory potential, weakening the normative-critical potential that characterised early interventions. In this context, the central challenge involves defining the contours of an *emancipatory* eco-social policy. How should such a policy look?

Rather than specifying concrete eco-social policies, in this article I want to contribute to answering this question, discussing how eco-social policy relates to capitalism. I do so in four steps. In the first section, I consider the tendency in the literature on eco-social policy to avoid the concept of capitalism. Rather than explicitly problematising the latter, scholars usually question economic growth and develop ‘post-growth’ alternatives. While moving beyond growth is essential, I argue that post-growth discourses risk being co-opted by neoliberal elites. In the second section, I discuss Matteo Mandelli's influential definition of eco-social policies, showing how it can weaken the critical potential of this field of study. In the third section, I consider Nancy Fraser's recent theorisation of (anti-)capitalism, arguing that it could help us to delineate the political horizon for an emancipatory eco-social policy. Finally, in the fourth section, I discuss some dilemmas and challenges related to the realisation of this ideal, pointing to avenues for further research. Throughout the article, I consider social policy discourses not only in terms of their ‘scientific’ validity, but also in terms of their political consequences. Indeed, welfare discourses are not neutral, and they can either facilitate or hinder emancipatory social change, assigning a key ethical-political responsibility to social policy scholars (Laruffa, 2019).

## Post-growth without post-capitalism?

This section comprises two sub-segments. The first shows how the literature on sustainable welfare tends to problematise growth rather than capitalism, and the second discusses the risks that this might imply in terms of a technocratic co-optation by neoliberal elites.

### Problematising growth

The literature on eco-social policies is concerned largely with the question of economic growth. As Cucca et al. (2023: 16) argue, the debate can be simplified by distinguishing between those who support ‘green growth’ and those who embrace ‘degrowth’ (or post-growth) visions. Green growth relies on technological improvements to reconcile economic growth with ecological demands. In this context, social policies have to fulfil essentially two goals: offering social protection to workers losing their jobs in unsustainable sectors and investing in people's ‘human capital’, so that they can be included in the emerging ‘green economy’. While this is now the dominant policy approach to the ecological transition, the critical academic literature is based on the insight that economic growth cannot be ecologically sustainable and that we need to move to ‘post-growth’ welfare states (e.g., Dukelow and Murphy, 2022; Hirvilammi and Koch, 2020; Koch, 2022).

Strikingly, this literature tends to avoid explicit reference to capitalism, problematising economic growth instead. For example, in *Towards Sustainable Welfare States in Europe*, edited by Schoyen, Hvinden and Dotterud Leiren (2022), the word ‘capitalism’ appears only 24 times in an almost 300-page book, and, of these, capitalism is mentioned 22 times in a neutral/uncritical context, e.g., in the references (especially Hall and Soskice's ‘Varieties of *Capitalism*’). Similarly, from reviews of the literature on eco-social policy setting the future agenda of the field (Bohnenberger, 2023; Cotta, 2024; Hirvilammi et al., 2023; Snell et al., 2023), it emerges that scholars discuss the link between eco-social policy and growth but avoid problematising capitalism explicitly.

To be sure, theorists of sustainable welfare are deeply critical of capitalist modes of production and consumption. When they criticise growth, they clearly mean capitalist growth: they question growth as a measure of wellbeing, proposing alternative, needs-based conceptions of human flourishing. However, while early theorists of sustainable welfare such as Ian Gough and Max Koch discussed capitalism explicitly, scholars now often refrain from doing so. This choice can be motivated by different reasons. Milena Büchs – one of the leading scholars in the field – uses the concept of capitalism in her work (including in publications co-authored with Max Koch). She sees growth and capitalism as closely intertwined and, as a consequence, she considers that postgrowth positions are inevitably anti-capitalist. However, she also acknowledges that the term capitalism may be problematic. In an interview in which she was asked to reflect on the use of the term capitalism in debates on sustainable welfare, Büchs (2021b) argues that one problem is that capitalism is ‘a loaded term and often there isn't sufficient space to explain what theoretical baggage you agree or disagree with if you use that term'. In particular, once you employ the word capitalism, ‘people immediately assume you are a Marxist', and ‘when you say we need to abandon capitalism, people immediately think you are suggesting we need socialism or communism' – of the type of ‘the Cold War era'. Similarly, eluding the concept of capitalism can be a pragmatic choice for being listened to in mainstream circles and thus having an impact on policies.

There might be another connected but slightly different reason for avoiding the concept of capitalism: that of building alliances with those who – while sharing the same values of sustainability and justice – are sceptical about the possibility of overcoming capitalism. These ‘realists’ are disillusioned with discourses that focus on alternatives to capitalism, which they discard as simply unfeasible. They focus on making capitalism more just and sustainable, and explicitly anti-capitalist discourses might frighten them. Rather than at the normative level, these disagreements concern the epistemic-ontological question of what in society can be realistically changed and what instead should be accepted as given. From this perspective, a discourse centered on post-growth might speak to people who are less critical of capitalism or who have difficulties in identifying with socialist ideals (including for reasons of pragmatism), thereby helping to build progressive coalitions across society.

However, problematising growth rather than capitalism could also have negative consequences. Lamenting a similar tendency to avoid the concept of capitalism in ecological economics, Pirgmaier and Steinberger (2019: 4) argue that this amounts to a ‘fetishization of growth as the core problem’, downplaying its ‘deeper-seated social drivers’. However, the problem is not ‘the economy’ in abstract terms – as a concern for economic growth might suggest – but *capitalism* as a historically specific class-based social system. A discourse that fails to explicitly present capitalism, rather than growth, as the core problem risks preventing an ‘open confrontation’ with the institutions that impede sustainability transformations, marginalising questions of ‘agency’ and ‘power’ (Pirgmaier and Steinberger, 2019: 5). Indeed, capitalism describes a type of society that ‘authorises an officially designated economy to pile up monetised value for investors and owners, while devouring the non-economised wealth of everyone else’ (Fraser, 2022: XV). This feature gives capital-owners a key responsibility in perpetuating both environmental unsustainability and various forms of injustice, as profits take precedence over eco-social concerns. Below, I show how a welfare discourse that does not explicitly problematise capitalism risks being technocratically co-opted by neoliberal elites.

### The risk of technocratic co-optation

Related to the problematisation of growth, the ideal eco-social policy is formulated in terms of post-growth/degrowth (e.g., Dukelow and Murphy, 2022; Hirvilammi and Koch, 2020; Koch, 2022). For example, Schulze Waltrup (forhcoming) develops a typology of eco-social policies in terms of their ability to drive a social-ecological transformation. The central criterion for classifying eco-social policies is their relation to economic growth: the least transformative perspective is ‘green growth’, whereas the most transformative approach is ‘degrowth’. This is not the place for an in-depth discussion of the merits and shortcomings of the post-growth/degrowth concepts. However, it is true that while there is a clear difference between ‘post-growth’ and the absence of growth in a growth-based society – degrowth involves an entirely different society, whereas reducing growth in existing societies entails a recession, i.e., a social disaster – some confusion emerges from the fact that both its critics (Naudé, 2023) and its supporters (Jackson, 2021: 20−21) argue that post-growth is, at least in rich countries, already a reality. Indeed, many mainstream economists, including Larry Summers, have argued that low and declining growth might be the new normal in an age of ‘secular stagnation’ (Jackson, 2021: 21). This is precisely the point where degrowth risks becoming captured by contemporary capitalism: post-growth is then transformed into something similar to neoliberal retrenchment. Through a political discourse of resource scarcity, the claim can be made that the good old times of ‘luxury’ consumption are over, leaving the poor to pay the bill of ecological adaptation. In terms of eco-social policies, there is a risk of selective implementation of misconceived degrowth theories, e.g., providing ‘universal basic services’ of bad quality, while promoting austerity measures based on a misinterpretation of two post-growth arguments: that we need to reduce consumption for ecological reasons; and that true wellbeing is disconnected from material opulence. Thus, a de-politicised and moralised understanding of degrowth can lead to a modernisation and stabilisation of neoliberal capitalism (Schoppek, 2020).

On this basis, I argue that the central risk of welfare discourses that problematise growth rather than capitalism is that they can be framed more easily in moralised and de-politicised terms than anti-capitalism. On the one hand, moralisation occurs when degrowth is framed in a way that does not confront capitalist power, reducing it to an individual lifestyle and consumption practice. In contrast, the concept of capitalism problematises production, rather than consumers’ behaviour, thereby questioning firstly the power asymmetry between capital-owners and the rest of society. On the other hand, de-politicisation arises when the problematisation of growth is disguised as a technical matter of finding better indicators – an issue of measurement. While the persistence of GDP as an indicator of social progress can be explained by the fact that it better serves, with respect to alternative wellbeing indicators, the goals of capitalist economies (Felice, 2016), explicitly problematising capitalism rather than growth would address the root problem rather than the symptoms. Thus, the advantage of the concept of capitalism is that it points openly to a specific kind of social relations as the root causes of injustice and ecological degradation, thereby allowing the debate to be framed in political terms. Adopting a political-relational and conflictual approach, the concept of capitalism calls for the mobilisation of social forces to redistribute power in society, i.e., to *change social relations* (e.g., Foster, 2002: 101−102), e.g., modifying property structures and investment decisions (e.g., Löwy, 2015).

To be sure, the problem is not with post-growth/degrowth theories but with the *technocratic co-optation* of these theories by neoliberal elites. Co-optation is the shift of meaning of a concept that subordinates its original meaning to a different political priority: in this process, ‘the concept itself is not rejected, but its initial meaning is transformed and used in the policy discourse for a different purpose from the original one’ (Stratigaki, 2004: 36). This creates two problems. First, transforming the meaning of a concept could lead gradually to its policy impact deteriorating, potentially even producing a negative outcome. Second, co-optation ‘works against mobilisation and pressure by interested parties and individuals by using the original as well as the transformed meaning as an alibi’: ‘It is difficult to mobilise against a claim that appears to be one's “own” even if it is no longer used to mean what one intended’ (Stratigaki, 2004: 36).

This is precisely what is happening with post-growth ideas. For example, in May 2023 the European Parliament organised the ‘Beyond Growth’ conference. In the statement opening the conference, the president of the European Commission, Ursula von der Leyen (2023), argued that ‘a growth model centred on fossil fuels is simply obsolete’ and that ‘we need to decarbonise our economies as quickly as possible’. For von der Leyen, going ‘beyond growth’ means replacing the current model of growth with a new one: the European Green Deal involves a new ‘growth model’ to become ‘the first climate neutral continent’ and bring about ‘a systematic modernisation of Europe's industry’. In this model, the economy needs to be made sustainable through technological innovation and the shift to renewable energy, ‘because in the long run, only a sustainable economy can be a strong economy’. After citing Robert Kennedy's famous critique of GDP, her speech ends with a reminder that economic growth ‘is not an end in itself’ and that ‘growth must not destroy its own foundations’. The goal of the European Commission seems that of transforming a radical critique of growth itself into a critique of a certain kind of growth, to be replaced by another ‘sustainable’ and ‘social’ type, which, in the long term, is even more profitable than its predecessor. While the European Green Deal might contain some minimal ‘degrowth’ elements (Ossewaarde and Ossewaarde-Lowtoo, 2020), the goal is to incorporate some of the ‘counterhegemonic narratives’ to eliminate the antagonism, thereby closing down any opposition (Samper et al., 2021: 14).

## Reducing the critical potential of a concept to make it applicable empirically?

In this section, I discuss Mandelli's definition of eco-social policies (Mandelli, 2022). While the literature on eco-social policy discussed so far is largely critical of economic growth, Mandelli's approach takes a more benevolent view on ‘green growth’, threatening to weaken the critical potential of the sustainable welfare discourse that has dominated debates on eco-social policy until now. Despite the fact that Mandelli's definition is recent, it has become rapidly influential in the field – including among post-growth advocates (e.g., Murphy, 2023) – and it is likely to become a point of reference in the literature. For this reason, I think it is important to scrutinise it carefully. The next section comprises three sub-segments: the first presents Mandelli's definition of eco-social policies, and the second and third formulate a sympathetic critique of the former.

### Re-defining eco-social policies

Mandelli (2022) discusses eco-social policies in the context of three distinct spheres, each with its own intrinsic goals. The social sphere is the sphere of the welfare state (comprising social insurance, social assistance, public services and labour market policies), which aims to (re)distribute resources, insure against social risks and guarantee people a decent standard of living independent of their participation in employment. The environmental sphere aims to preserve the natural environment. The purpose of the economic sphere is to promote economic growth, i.e., to increase ‘production, consumption and exchange of goods and services in the market’ (Mandelli, 2022: 336).

Against this background, different eco-social policies are conceivable, according to whether and how (i.e., positively or negatively) social, environmental and economic goals are related and to whether these three goals should be governed in ‘silos’, ‘hierarchically ordered’ or ‘integrated’ (Mandelli, 2022: 337). There are three possible kinds of links between these goals: 1) ‘neutrality’, where the goals are unrelated; 2) ‘synergy’, where the promotion of one goal automatically also promotes the other goals; and 3) ‘trade-off’, where the promotion of one goal is inversely related to the promotion of the other goals. To the extent that the goals are seen as unrelated, a ‘silo logic’ to policymaking prevails, as the goals are pursued independently of the others. Hierarchical ordering occurs when goals are connected by an unsolvable trade-off. Finally, policy integration happens either in cases where goals are related positively by default or in cases where a trade-off between the goals is seen as at least partly solvable or avoidable. In this latter case, ‘synergies do not arise automatically, but can be achieved through policy integration’ (Mandelli, 2022: 337). Approaches involving policy integration assume that solving the ‘eco-social-growth trilemma’ is both feasible and desirable: these approaches – most prominently exemplified by the ‘sustainable development’ agenda promoted by the United Nations – are ‘balanced’ because they aim to ‘reconcile social, environmental and economic objectives’. In contrast, post-growth approaches do not aspire to solve the eco-social-growth trilemma: questioning the desirability of economic growth, they aim rather to establish a hierarchical ordering, where economic growth is de-prioritised to the benefit of eco-social goals (Mandelli, 2022: 338).

Mandelli (2022: 339) is concerned with the ‘widespread tendency towards normativity’ in the field of eco-social policy, where scholars are ‘more interested in prescribing which policies should be pursued, rather than describing what they are’: eco-social policies ‘are most often defined in light of their ability to achieve certain outcomes, often relying on post-growth approaches’, thereby failing to identify existing eco-social policies, such as those based on balanced approaches. In order to provide an empirically applicable description of eco-social policies, Mandelli (2022: 340) proposes to define them as ‘public policies *explicitly* pursuing both environmental and social policy goals in an *integrated* way’ (emphasis in original). He wants this definition to be a ‘non-normative conceptualisation’: rejecting the ‘outcome-based’ approach of current definitions of eco-social policies, which ‘evaluate policies in light of their expected social and environmental impacts’, this definition is based on the ‘declared objectives’ of the policy. The goal is to facilitate ‘policy analysis’. On the one hand, this definition avoids the confusion that arises from normative-oriented approaches, where ‘certain policies might be considered eco-social by some and not by others, depending on their own normative definitions of desirable policy outcomes’. On the other hand, this definition also includes those policies that are unsuccessful in reaching socio-ecological outcomes, i.e., empirical studies can now identify and evaluate eco-social policies, ‘even when their outcomes are deficient’ (Mandelli, 2022: 340). In this framework, ‘even growth-oriented policies can be defined as eco-social’ (Mandelli, 2022: 343).

In the following section, I provide a sympathetic critique of this framework. Mandelli seeks to improve research in the field of eco-social policies, and my goal here is not to ‘demolish’ his valuable efforts to advance the clarity and precision of the work done in this field. Rather, my aim is to point out some of the potential pitfalls of Mandelli's approach, especially once a critical perspective on social policy is embraced. My central concern is that Mandelli's definition might be too broad and too weak, facilitating a de-politicised economisation of eco-social goals, i.e., the subordination of society and nature to the profit-driven economy. Considering green growth as part of eco-social policies, his approach takes a step backward with respect to the literature on sustainable welfare, which affirms vigorously the need for a radical social-ecological transformation of current socioeconomic systems. While this literature clearly adopts a *hierarchical* approach, in which economic goals are subordinated to eco-social aims, Mandelli's definition considers eco-social policies to also include those that aim to reconcile economic, ecological and social goals, treating them as equally important and maybe even those that prioritise economic objectives. I articulate my sympathetic critique of Mandelli's framework around two points: the first relates to the relationship between the normative and the empirical aspects of defining eco-social policies, and the second derives from his discussion of the three spheres.

### A weak normative content

The first weakness of Mandelli's approach is that it relies on a false dichotomy between normativity and empirical applicability. According to his framework, it is problematic that definitions of eco-social policies have normative content. With a view to facilitating empirical analyses, he thus proposes a ‘non-normative conceptualisation’. However, this way of proceeding assumes wrongly that in order to make a concept empirically relevant, we need to first eliminate its normative content. Instead, it is perfectly possible to evaluate reality empirically against a normative yardstick – and indeed this is what critical social policy scholars often do. Proposing definitions that describe the *ideal* of eco-social policies is important, because, even if the latter is never fully achieved in reality, it sets a standard against which to evaluate real-life policies. As Crouch (2004: 3) argues, it is always valuable to consider ‘where our conduct stands in relation to an ideal, since in that way we can try to improve’, and it is essential to take this approach rather than the more common one, ‘which is to scale down definitions of the ideal so that they conform to what we easily achieve’, because that way promotes only ‘self-congratulation’, neglecting how social reality deteriorates.

According to Mandelli's framework, we should reject ideal normative criteria: eco-social policies are those that are regarded as such by the actors promoting them. However, the failure of sustainability policies lies precisely in the fact that they are usually sustainable only at the rhetorical level. For Mandelli, the dominant growth-based approaches count as eco-social policies. However, empirical evidence suggests that green growth is not actually possible. Very often, ‘green’ policies actually simply displace their social and environmental costs elsewhere. For example, to the extent that moving to a low-carbon economy in the Global North requires the production of batteries and solar panels, the ecological transition in the Global North risks relying on forms of neo-colonialism, forcing a massive expansion in the extraction of minerals and metals in the Global South, intensifying social and environmental injustice there (Bainton et al., 2021). Without a normatively ambitious definition of eco-social policy – one that, for example, takes the global dimension of eco-social justice into account – the danger is that when examining the eco-social policies of the European Union (EU), it can be overlooked that they entail the ‘greening of the Empire’ (Vela Almeida et al., 2023). As Brand and Wissen (2021) make clear, the ‘mode of living’ of populations in the Global North is intrinsically ‘imperial’, i.e., dependent on an ‘elsewhere’ in which the costs of its unsustainability are imposed in terms of resource extraction, waste exports, externalisation of production and thus of pollution. Clearly, eco-social policies should alter this mode of living in the Global North. Instead, the EU's goal of becoming the first climate-neutral continent seems neo-mercantilist: sustainability, like social policy in ‘social investment’, must become a productive factor and an asset in international competition (Laruffa and Nullmeier, forhcoming).

However, these mainstream ‘balanced eco-social policies’ are also weakly emancipatory for the population in the Global North. As Velicu and Barca (2020) argue, the prevailing approach to a just transition is based on undisputed social categories: it fails to question oppressive identities generated by existing institutions and the hierarchies they create, such as the subaltern position of workers within capitalism. A more emancipatory approach would demand the democratisation of work, allowing workers themselves to ‘reimagine the future of society they want and to design the transition’ (Velicu and Barca, 2020: 264). In this more radical perspective, justice ‘does not revolve solely around demanding better wages and labour conditions’ but also around workers’ freedom to be recognised as something other than wage-workers, ‘to speak for themselves, and to give themselves another name’, overcoming an unjust ‘recognition order’ (Velicu and Barca, 2020: 268). A just transition should make it possible ‘to live in a different type of society, not simply a low-carbon version of the current one’ (Healy and Barry, 2017: 453). Seen from this perspective, the attempt to establish a ‘non-normative’ definition of eco-social policies turns out to be highly – even if hiddenly – normative: legitimising green growth, it marginalises more emancipatory alternatives.

### The problematic logic of the three spheres

Mandelli discusses eco-social policies in terms of three ‘spheres’: the ‘social’, the ‘environmental’ and the ‘economic’. While he briefly mentions that the sphere of politics could be added as a fourth sphere, he focuses the discussion on the three other spheres. The choice of excluding politics from the framework is significant, as it denies the political nature of eco-social policies, leading to the adoption of a technocratic approach to welfare reform. Balancing social, ecological and economic goals is transformed into a de-politicised exercise: once society is emptied of its political content − that is, of the power relations on which it is based − moving to a sustainable society becomes a matter to be managed in a technocratic way, i.e., a task that does not need democratic involvement or discussion and that can be delegated easily to the ‘experts’ of national and international agencies. However, it is precisely the political sphere that would allow power asymmetries in society to be changed, permitting social transformation. Deciding how to articulate eco-social and economic goals should be a democratic matter, as this is the only way to overcome the deep inequalities that characterise the status quo. Currently, priority is given to economic objectives over eco-social aims, because this hierarchy promotes the interests of the dominant actors in the political sphere. An emancipatory approach to eco-social policy would point to the need to reinforce the participation in the political sphere of those groups that are excluded, oppressed and silenced by the current system, with a view to challenging the existing hierarchy between economic and eco-social aims.

A further problem that emerges from this framework is the unintended acceptance of a profit-oriented economic sphere indifferent to any eco-social rationality. The tasks of realising social and ecological goals are delegated to the respective spheres, de-politicising the capitalist logic of profit-making. To the extent that eco-social goals are promoted, they must first be reframed as economic goals: nature is conceived as ‘natural capital’, society as ‘social capital’, individuals as ‘human capital’ and social policy as ‘social investment’. Instead, an emancipatory strategy would require the economic sphere to be eco-socially responsible. Rather than putting human beings and non-human nature at the service of the economy and profits, the goal would be to put the economy at the service of human and ecological wellbeing.

The very language of the ‘spheres’ is already problematic, as it suggests that the economy can be separated from society and the natural environment. Karl Polanyi's arguments on the ‘embeddedness’ of the economy might be helpful here, as they imply that it makes no sense to speak of an autonomous economic sphere with internal laws of functioning. Economic relations *are* social relations, which also depend inevitably on the natural environment of which society is part. On this basis, Polanyi (2001) argues that there are only two possibilities: either the economy is subordinated to eco-social goals or vice-versa. While capitalism is the aberrant system in which society and nature are treated as mere adjuncts of the economy, socialism entails the subordination of the economy to a democratic society.

Against this background, it appears evident that the ‘trilemma’ that Mandelli identifies is not inscribed in the laws of nature but is rather the specific result of the historically contingent socioeconomic system called capitalism, i.e., a system in which the economy is oriented towards profits rather than eco-social needs. As Barca and Leonardi (2018: 488) show with respect to the job-environment dilemma faced by workers, the right solution is to reject this perverse choice altogether, politicising and reframing the economy from below. This approach aims to subordinate economic production to social reproduction, prioritising use-value over exchange-value, while affirming that citizens and workers should be the people who decide what should be produced and how (Barca and Leonardi, 2018: 498).

**Figure 1.**
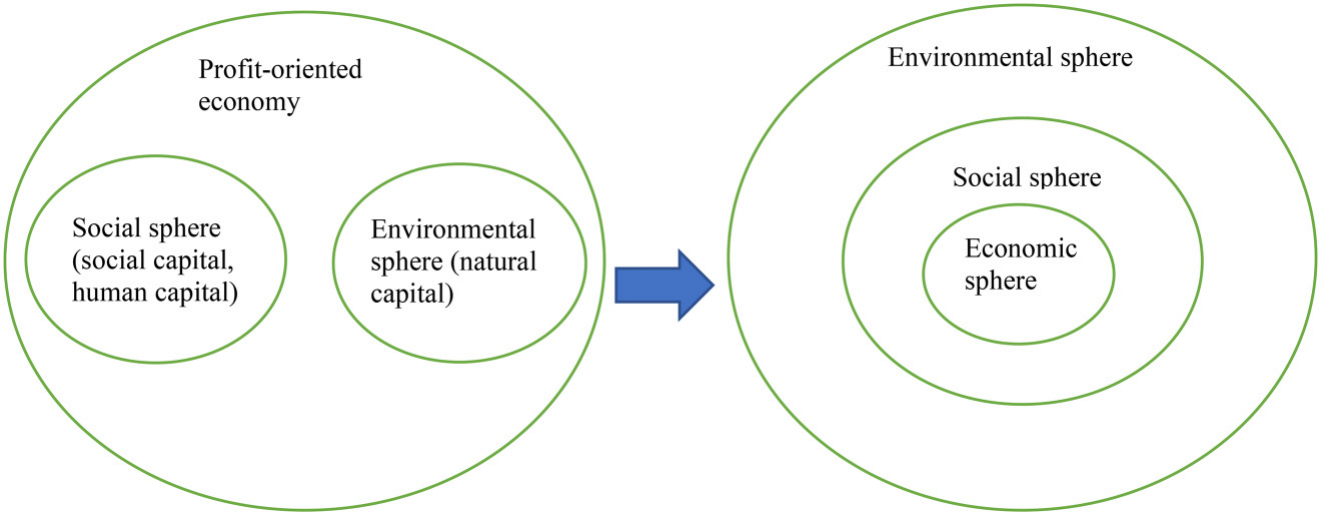
From a policy approach that reconciles economic, social and ecological goals through economisation, to a policy approach that acknowledges the ‘embedded’ nature of the economy and thus subordinates the latter to an eco-social rationale.

Source: Author's own elaboration.

Rather than three separate spheres in need of reconciliation – mainly through the economisation of eco-social goals – the ‘embedded-economy’ perspective suggests considering economic relations as a subset of social relations and the latter as part of the natural environment, i.e., three concentric spheres, where the ‘economy’ is subordinated to an eco-social rationale (Figure 1).

## Capitalism versus socialism?

The discussion so far has highlighted the absence of an explicit problematisation of capitalism in the literature on eco-social policy. While the literature on ‘sustainable welfare’ tends to problematise growth rather than capitalism, more recent attempts to conceive eco-social policies in non-normative terms risk marginalising the critique of capitalism, legitimising those approaches that subordinate eco-social goals to economic growth. In this section, I discuss some advantages of an explicit problematisation of capitalism for eco-social policy debates. I am not suggesting a black-or-white approach, where all efforts to promote eco-social goals within capitalism are futile. Rather, I argue that eco-social policies should be framed in terms of their capacity to promote eco-social goals in a way that modifies the economy (Carrosio and De Vidovich, 2023) and helps to undermine capitalism. This is in line with recent efforts to critically consider the broader ‘global political economy’ within which eco-social policies are embedded (Schulze Waltrup et al., 2023). In this context, there is some evidence that scholars are now moving towards a more explicit critique of capitalism. For example, Nenning et al. (2023) explicitly discuss eco-social policies in terms of ‘anti-capitalism' and ‘eco-socialism'.

Understanding the relevance of anti-capitalism for eco-social policy requires going beyond a narrow class-based understanding of capitalism based on the exploitation of workers. For example, it demands recognising that colonialism and slavery were vital to the emergence of capitalism and that these forms of racial and colonial expropriation of land and labour continue today (e.g., Bhambra, 2021; Issar, 2021). Similarly, it requires acknowledging that, in separating economic production from social reproduction and in valuing the former over the latter, capitalism necessarily entails gender inequality and women's oppression (e.g., Bhattacharya, 2017; Bieler and Morton, 2021).

Therefore, I draw on Fraser's recent theorisation of capitalism as the root cause of all major crises that humanity faces: extreme inequality, widespread poverty and precarity; a shortage and undervaluation of care; racialised violence and international tensions and wars; ecological disaster; and powerless democracies (Fraser, 2022). All these crises converge, exacerbating one another, generating a crisis of the whole social order. Importantly, Fraser conceives capitalism not as a type of economy but as a type of society, bringing to the fore its non-economic preconditions. One of the central features of capitalism is that it constructs a separate ‘economic system’, treating ‘its structuring social relations *as if* they were economic’ (Fraser, 2022: 17, emphasis in original). Capitalism thus hides the ‘non-economic background conditions’ that make possible a profit-oriented economic system. These conditions include: the expropriation of colonised and racialised subjects (such as slaves and ‘illegal’ migrants); the unpaid care work performed mainly by women within families; the exploitation of natural resources; and public infrastructure (e.g., repressive forces that enforce order, legal systems that guarantee private property rights and multiple public infrastructures that enable private firms to function profitably). While capitalism necessarily relies on these conditions, it also undervalues them, limiting their inherent emancipatory potential. In this account, capitalism is *structurally* racist, patriarchal, ecologically unsustainable and undemocratic – a conceptualisation that brings together in a single frame all the oppressions and contradictions of the present conjuncture.

Related to this diagnosis, the solution is to coordinate the struggles of multiple social movements and various collective actors with a view to building an emancipatory counterhegemonic project of eco-social transformation. Especially relevant for the purpose of this article is the argument that this requires overcoming ‘single-issue environmentalism’, which inevitably becomes an ‘environmentalism of the rich’ that intensifies ‘off-shore extractivism’, to embrace instead a ‘social’ and ‘anti-colonial’ environmentalism, or, more simply, ‘ecosocialism’ (Fraser, 2022: 109). In this perspective, ‘we cannot save the planet without disabling some core, defining features of our social order’: ecopolitics ‘must, in sum, be anti-capitalist’ (Fraser, 2022: 85). We have to reverse priorities: while capitalism subordinates the demands of ‘social, political and ecological reproduction’ to the imperative of profit-maximisation, ‘socialists need to turn things right side up – to install the nurturing of people, the safeguarding of nature, and democratic self-rule as society's highest priorities’ (Fraser, 2022: 152). In short, ‘socialism must put squarely in the foreground those matters that capital relegates to its disavowed background’ (Fraser, 2022: 152).

Of course, there is a large body of literature on ecosocialism (and eco-Marxism), eco-feminism and an anti-colonial perspective on ecology that shares these fundamental insights. What matters is that this theorisation of (anti-)capitalism might provide the normative-political horizon for an emancipatory definition of eco-social policies. While a democratic, feminist, anti-racist/anti-colonial eco-socialism represents an (unachievable?) ideal, it remains useful to orient and evaluate real-life policies. This definition thus follows the ‘utopian’ approach to social policy described by Levitas (2001: 450) in this Journal, which consists of thinking ‘first about where we want to be, and then about how we might get there’. Eco-social policies are those that make steps forward towards this vision, advancing eco-social goals in a way that makes the real world closer to the ideal while undermining the power of capital.

However, note that not only the eco-socialist ideal but also the capitalist commitment to endless accumulation is ‘utopian’. In fact, Polanyi (2001: 3) describes the capitalist project – one that subordinates nature and society to profit-oriented markets – as a ‘stark utopia’. The capitalist utopia is that of unlimited accumulation for the few with environmental destruction and social injustices as side effects – and this vision is arguably even less realistic than the eco-socialist project, which at least considers the finitude of resources. Capitalism and socialism can thus be viewed as two opposed utopian projects, and the two ideals delimit the terrain of eco-social politics: moving in the direction of one pole simultaneously implies a move away from the opposite pole.

The explicit opposition to capitalism might represent a novelty for social policy. Indeed, social policy and capitalism have a contradictory relationship. While social policies often embody non-capitalist values (e.g., distributing resources according to need), they are also congenial to capitalism at the ideological level (reinforcing its legitimacy) and at the functional level (e.g., stabilising consumers’ demand during economic downturns and sustaining a healthy and educated workforce). Obviously, it *is* possible to have more or less ‘social’ and ‘green’ versions of capitalism. Framing the field of eco-social policy as a continuum between the two ‘pure’ poles of capitalism and socialism implies that there are many in-between positions: in a predominantly capitalist society, there is nevertheless room to promote eco-social goals. However, if the insight that capitalist logic represents a necessary upper limit – a ‘ceiling’ – to our possibilities to realise eco-social goals is correct, an exclusive reformist approach will be largely insufficient. At some point, we must break with capitalism – move beyond the half-way of the continuum – and prioritise eco-social goals over profit-maximisation. However, the urgency of addressing the ecological crisis poses a real dilemma: we have no time to wait for the end of capitalism to start acting, but we do not have time for small-step, unambitious reformism, and we need radical-transformative solutions. As anti-capitalist parties are not winning elections in the Global North, how should critical scholars handle more pragmatic reforms that ameliorate the situation with respect to the status quo while possibly reinforcing capitalism, making the latter more ‘sustainable’ and ‘inclusive’?

## A research agenda for critical social policy

In the previous section, I argued that emancipatory eco-social policies are those that make steps towards the ideal of eco-socialism, moving away from the capitalist utopia of a world governed through profit-maximisation. The problem with the continuum idea is that it suggests a linear relationship between the promotion of eco-social goals and advancements towards eco-socialism. However, in reality, things might be more complex. Indeed, it is possible that a trade-off – rather than a synergy – exists between the promotion of eco-social goals within capitalism and a move towards eco-socialism. Policies attempting to promote eco-social goals might do so through the *reinforcement* of key capitalist logics, such as productivism. A ‘green’, jobs-rich version of economic growth is surely better than an exclusionary and environmentally destructive version. However, these ‘solutions’, which are largely insufficient to address current eco-social challenges, are not merely pragmatic steps in the direction of a deeper social-ecological transformation: they often go precisely in the opposite direction, further commodifying human and natural ‘capital’ alike. These reflections echo a broader dilemma that scholars face: while adopting technocratic-economised discourses might allow welfare scholars to influence social policy choices in a progressive direction, embracing politicised-oppositional discourses could relegate them to marginal positions, making it difficult to have an impact on policy (Laruffa, 2019). In this context, the fact that the critique of economic growth can be framed within a technocratic approach is not only a disadvantage but also an advantage (a conference on anti-capitalism chaired by the President of the European Commission is hard to imagine).

From this perspective, the key question becomes the following: How can we move away from capitalism while simultaneously promoting eco-social goals in the here and now? What are the criteria for *non*-reformist eco-social reforms that, while improving the situation within the current system, could also bring about a deeper social-ecological transformation? How can we distinguish these reforms from those that, while improving eco-social outcomes, further entrench capitalist logic in society, making it harder to overcome capitalism in the long term? This is an area where more research by critical social policy scholars is needed.

For example, Gough (2022) identifies two scenarios for eco-social policies. The first entails a ‘Green Deal’ aimed at substantially raising ‘green’ spending (both private and public) while securing minimum levels of wellbeing through the collective provision of essential goods and services, involving policies such as universal basic services, job guarantees (especially promoting ‘essential’ jobs in the ‘foundational economy’), fair wages and a guaranteed minimum income. This scenario, centred on *fair* green growth, ‘would reverse the neo-liberal austerity project of the last decade but would not be incompatible with emerging trends in contemporary capitalism’ (Gough, 2022: 470). The second scenario entails policies for an ‘economy of egalitarian sufficiency’. Recognising the extensive and urgent obligations of rich countries to contribute to decarbonisation on a global scale, this scenario would tackle unsustainable consumption patterns in a fair way (Gough, 2022: 470). In this scenario, social policies not only redistribute income and wealth but also ‘recompose consumption’ (Gough, 2022: 465) with a view to promoting a ‘need-based economy’ through policies such as the implementation of ceilings on income and wealth; the prohibition, regulation and taxation of luxury and wasteful consumption; the expansion of essential public employment and shrinking of destructive jobs; and a cutback of advertising (Gough, 2022: 468−469). Even if these policies could be implemented gradually and in a reformist manner, they are clearly part of an anti-capitalist project, as they openly oppose capitalist interests and demands. While Gough does not use the concept, a ‘need-based economy’ is evidently a socialist, not a capitalist, economy. What Gough does not discuss, however, is the relationship between the two scenarios. Do they differ in merely ‘quantitative’ terms? Are the two scenarios positioned on a continuum, where a stronger and deeper implementation of the first leads quasi-automatically to the second? Or are the ‘qualitative’ differences so profound that they create an unsurpassable discontinuity between the two? In other words, would the implementation of the first scenario, which is (more or less) compatible with ‘green capitalism’, facilitate the implementation of the second, eco-socialist project? Or would the first scenario stabilise ‘green capitalism’ in ways that make it harder to realise the eco-socialist vision? The discussion undertaken in this article suggests that critical social policy scholars should be attentive to these questions, when assessing real-world eco-social policies.

## Conclusion

Starting with the assumption that scholars’ theories and discourses are not neutral but politically performative and thus require reflexivity, this article has discussed the absence of an explicit problematisation of capitalism in the literature on eco-social policies (although this might be changing now). Heterodox scholars have focused largely on criticising growth rather than capitalism, but neoliberal elites have started to use misconceived post-growth/degrowth theories for their own purposes: austerity and green capitalism. Recent ‘non-normative’ conceptualisations of eco-social policies – are these possible? – risk legitimising policies that subordinate eco-social goals to growth, further marginalising an explicit critique of capitalism. I proposed a normatively rich, empirically applicable definition of eco-social policies as those that prioritise eco-social goals over profits, suggesting that a feminist, democratic, anti-racist/anti-colonial eco-socialism should provide the ultimate normative horizon for critical social policy scholars’ conceptualisation of eco-social policies: the final goal of an emancipatory politics in the twenty-first century should be that of overcoming capitalism, subordinating the global economy to social-ecological needs rather than profits. While this objective might involve different strategies, it is essential that critical social policy scholars do not lose sight of this vision. However, accepting this ambition does not resolve all problems, as an explicit anti-capitalist approach could fail to influence policy choices. Addressing this dilemma requires progressive forces, including critical social policy scholars, to embrace a double strategy, which seeks to advance concrete reforms within the currently dominant system *and* to transcend the latter, i.e., to identify those reforms that, while advancing eco-social goals within capitalism, also help to surpass it. While more research is needed on these matters, this perspective demands that scholars engage not only in the top-down development of progressive eco-social policies. Interacting with civil society and social movements through various practices of scholar-activism, critical academics should also aim at bottom-up consciousness-raising, building counterculture and counterpower based on the insight that a fully sustainable and just capitalism remains an illusion.
